# Mepolizumab for the management of chronic rhinosinusitis with nasal polyps across the United States: A retrospective study

**DOI:** 10.1016/j.jacig.2025.100549

**Published:** 2025-07-31

**Authors:** Juan Carlos Cardet, Jared Silver, Martin Maldonado-Puebla, François Laliberté, Chi Gao, Ramya Ramasubramanian, Annalise Hilts, Kaixin Zhang, Jeremiah Hwee, Waseem Ahmed, Amy G. Edgecomb

**Affiliations:** a Department of Internal Medicine, Division of Allergy and Immunology, University of South Florida Morsani College of Medicine, Tampa, Fla; b US Medical Affairs–Respiratory, GSK, Durham, NC; c Groupe d’analyse, Ltée, Montreal, Quebec, Canada; d Analysis Group Inc, Boston, Mass; e Analysis Group Inc, Los Angeles, Calif; f Global Epidemiology, GSK, Mississauga, Ontario, Canada; g Global Data Generation, GSK, London, United Kingdom; h Anti-Infectives and Respiratory Business, US Value, Evidence, and Outcomes, GSK, Philadelphia, Pa

**Keywords:** Chronic rhinosinusitis with nasal polyps, nasal polyposis, claims-based data, mepolizumab, oral corticosteroids, nasal polyp–related treatments, sinus surgery, health care resource utilization, costs

## Abstract

**Background:**

Retrospective data are limited on the effectiveness of mepolizumab treatment that is reflective of real-world practice in patients with chronic rhinosinusitis with nasal polyps (CRSwNP).

**Objective:**

We evaluated changes in administration of nasal polyp (NP)-related oral corticosteroids (OCS) and other treatments, exacerbations, sinus surgeries, NP-related health care resource utilization, and costs before and after mepolizumab initiation in patients with CRSwNP.

**Methods:**

Retrospective cohort study using data from the Komodo Research database included adults with CRSwNP, without severe asthma, initiating mepolizumab therapy on or after July 29, 2021 (index date), with 12 months of continuous health care enrollment before index and ≥6 months after index. Treatment with reslizumab, benralizumab, or tezepelumab during the study period was excluded. Outcomes were compared pre- versus post-mepolizumab initiation for the overall population and on-label subgroup analysis.

**Results:**

Mean number of NP-related OCS dispensings per patient per year (PPPY) (0.5 after vs 1.2 before therapy initiation), total mean OCS dose (119.1 vs 309.9 mg), mean daily OCS dose per period (0.3 vs 0.8 mg), and mean number of OCS bursts (0.3 vs 0.7) were significantly lower after versus before initiation, respectively (all *P* < .001). Numbers of patients requiring NP-related treatments significantly decreased (75.0% post vs 86.7% pre, *P* < .001). Mean number of NP-related exacerbations experienced by patients significantly reduced (1.6 PPPY pre vs 0.3 PPPY post). Mean number of sinus surgeries significantly reduced after initiation (annual rate ratio [95% confidence interval] 0.23 [0.13, 0.40], *P* < .001), as did rate of otolaryngologist visits PPPY, excluding mepolizumab administration visits (rate ratio 0.52 [95% confidence interval 0.43, 0.63], *P* < .001).

**Conclusion:**

In this first retrospective mepolizumab study for patients with CRSwNP without severe asthma, improvements in all outcomes were observed after mepolizumab initiation.

Chronic rhinosinusitis with nasal polyps (CRSwNP) is characterized by chronic inflammation of the nasal mucosa and paranasal sinuses,^1^ affecting approximately 1.1% of the US population.^2^ Predominantly driven by type 2 inflammation, IL-5 is a key driver of disease pathogenesis.^3^ Primary symptoms include nasal congestion, rhinorrhea, and hyposmia.^1^ CRSwNP is associated with many comorbidities also driven by type 2 inflammation,^1^ such as comorbid asthma^2^ and allergic rhinitis,^4^ which affect 40% to 67% and 17% to 75% of patients with CRSwNP, respectively. The standard of care to manage symptoms of CRSwNP involves therapy with intranasal (INCS) and systemic corticosteroids (SCS)^5^^,^^6^ and/or sinonasal surgery.^1^ In times of CRSwNP^7^ symptom exacerbation, short, high-dose courses of oral corticosteroids (OCS) are deployed; however, 4 courses over a lifetime (0.5 to <1 g) are sufficient to cause serious negative effects such as osteoporosis, weight gain, and increased risk of infections.^8^^,^^9^ Associated comorbidities contribute to the high clinical burden (including steroid treatments and sinus surgeries) and health care costs linked with CRSwNP.^10^ Notably, incremental costs were $4,409 higher for patients with CRSwNP and comorbid asthma than for those without comorbid asthma; $4,502 higher for patients with CRSwNP treated with OCS than for those not treated with OCS; and $13,532 higher for patients with CRSwNP who have undergone nasal surgery than for those who have not undergone nasal surgery.^10^ Consequently, CRSwNP is associated with substantial symptom, treatment, and economic burdens, which negatively impact the patient’s quality of life.^1^^,^^11^

Mepolizumab is a monoclonal antibody that specifically targets IL-5, thereby inhibiting the downstream signaling pathway and reducing eosinophilic inflammation and symptoms of type 2 inflammatory diseases.^3^^,^^12^ SYNAPSE (NCT03085797) was a phase 3, randomized, placebo-controlled, multicenter trial that investigated the efficacy and safety of mepolizumab. Eligible patients were diagnosed with bilateral nasal polyps (NP), had severe NP symptoms, and had undergone ≥1 nasal surgery. Participants were randomized 1:1 to receive mepolizumab or placebo every 4 weeks, alongside standard of care with INCS, saline nasal irrigation, SCS, or antibiotics. Mepolizumab was found to improve nasal symptoms and health-related quality of life and to reduce NP size, number of nasal surgeries, and SCS receipt in patients with CRSwNP.^3^ Phase 3 randomized clinical trial settings are designed to test for efficacy of a medicinal product, but often with limited generalizability. Retrospective studies reflective of real-world practice are crucial to establish treatment effectiveness in settings outside of clinical trials. Previous retrospective studies among patients with CRSwNP and comorbid severe asthma have supported the receipt of biologics, including but not limited to mepolizumab.^13^, ^14^, ^15^, ^16^

However, these studies examined outcomes that did not distinguish whether SCS were used to treat severe asthma or CRSwNP. Therefore, there are few retrospective data reflective of real-world practice evaluating mepolizumab treatment on NP-related treatment outcomes specifically, as well as data regarding OCS receipt, health care resource utilization (HCRU), and costs in patients with CRSwNP without severe asthma.^4^ This study aimed to evaluate CRSwNP-specific outcomes before versus after initiation of mepolizumab treatment for patients with CRSwNP without comorbid severe asthma.

## Methods

### Study design

This was a retrospective cohort study (GSK ID:218957) of deidentified medical and prescription records from a large US all-payer medical and pharmacy claims database (Komodo Research database). No patient contact occurred, results were tabular, and analyses omitted subject identification. Consequently, informed consent, ethics committee, or institutional review board approval were not required. The database included data from ≥320 million patients enrolled in US health plans from 2012 to the present day, and encounters or claims were detailed from ≥150 payers, including Medicaid, commercial, and Medicare insurers. Patients with CRSwNP without comorbid severe asthma initiating mepolizumab after July 29, 2021, were selected, with the first dispensing or administration defining the index date. The pre-mepolizumab period was defined as the 12 months of continuous health plan coverage before mepolizumab initiation. The post-mepolizumab period spanned from 6 months after index until the earliest of either the end of continuous health plan coverage or the end of data availability (June 30, 2023).

### Patient population

Patients initiating mepolizumab on or after July 29, 2021, were aged ≥18 years, with separate diagnoses of both CRS and NP (using International Classification of Diseases, Tenth Revision, Clinical Modification [ICD-10-CM] codes J32.xx and J33.xx, respectively), during the pre-mepolizumab period or on index date. Patients received ≥2 doses of mepolizumab within 6 months of the index date and had continuous health plan enrollment 12 months before index (pre-mepolizumab period) and ≥6 months after the index date (post-mepolizumab period). Patients with ≥1 medical or pharmacy claim for reslizumab, benralizumab, or tezepelumab or with a diagnosis of severe asthma at any time during the study period (July 29, 2021, to June 30, 2023) were excluded, as were patients with ≥1 medical or pharmacy claim for mepolizumab before the index date. Given that reslizumab, benralizumab, and tezepelumab are biologics approved for severe asthma and not CRSwNP, the decision to exclude patients receiving these drugs supported the selection of the specified patient population.

Additionally, a subgroup of patients meeting the approved label criteria for treatment with mepolizumab (on-label population) were included as a separate subgroup analysis. Patients eligible for this subgroup had been dispensed or administered INCS in the pre-mepolizumab period or on the index date (as determined by generic product identifier or Healthcare Common Procedure Coding System codes) and were excluded if diagnosed with allergic fungal rhinosinusitis (ICD-10-CM codes J30.89 [allergic rhinitis] and B49.x [mycosis]) or cystic fibrosis (ICD-10-CM code E84.x) at any time during the study period. Patients otherwise meeting the eligibility criteria but who also had a severe asthma diagnosis (ICD-10-CM code J45.5x) during the pre-mepolizumab period or on the index date were included and analyzed in a separate comorbid severe asthma subgroup.

### Outcomes

Study outcomes were evaluated before and after mepolizumab initiation for the overall population and each subgroup separately. Outcomes included NP-related OCS receipt, treatments, and exacerbations; sinus surgery; and NP-related HCRU and health care costs. Additionally, composite end points—defined as (1) having either ≥1 OCS dispensed, sinus surgery, or a claim for NP-related antibiotic receipt and (2) having all 3 outcomes—that is, ≥1 OCS dispensed, sinus surgery, and a claim for NP-related antibiotic—were evaluated and compared before and after mepolizumab initiation among the on-label subgroup.

*NP-related OCS receipt* was defined as an inpatient or outpatient claim with a diagnosis of NP in the primary position and without a diagnosis of asthma within ±5 days of the service date for the OCS claim, or a medical claim for NP surgery within ±30 days of the service days for the OCS claim. *NP-related exacerbations* were defined as a medical claim for NP, without an asthma diagnosis within ±5 days of the NP diagnosis, and with one of the following within ±5 days of the NP diagnosis: ≥1 pharmacy claim for OCS with 7 to 21 days’ supply, increase in prednisone equivalent OCS dose, ≥1 pharmacy claim for antibiotics, or ≥1 medical claim for an endoscopy or polypectomy. *NP-related treatment* was assessed at the drug class level including INCS, leukotriene receptor antagonists, nasal saline, nasal saline and topical steroid combinations, and steroid-eluting nasal stents. NP-related antibiotic receipt was defined as an inpatient claim with a diagnosis of NP in the primary position within ±5 days without a diagnosis of asthma within ±5 days of the service date for the antibiotic claim, or an outpatient claim with a diagnosis of NP in any position without a diagnosis of asthma within ±5 days of the service date for the antibiotic claim, or a medical claim for NP surgery within ±30 days of the service day for the antibiotic claim. *Sinus surgeries,* including functional endoscopy sinus surgery and sinusotomy, were identified by Current Procedural Terminology codes. In addition to analyzing sinus surgeries after mepolizumab initiation versus 1 year before, a sensitivity analysis of sinus surgery outcomes used all data available before mepolizumab initiation, beginning at the earliest of start of continuous eligibility or data availability.

*NP-related HCRU* was defined as any medical visits with a primary or secondary diagnosis of NP or an NP surgery; visits were reported including and excluding visits associated with mepolizumab administration. *NP-related health care costs* were defined as any medical visits with a primary or secondary diagnosis of NP or an NP surgery; *NP-related pharmacy costs* were defined as any claim associated with an NP-related treatment. Costs were reported including and excluding costs associated with mepolizumab administration. All costs were inflated to the 2023 US dollar using the medical component of the Consumer Price Index from the US Bureau of Labor Statistics.

### Statistical methods

Demographics and clinical characteristics were described using data from the 12-month pre-mepolizumab period up to the index date, using mean, standard deviation (SD), and median for continuous variables, and frequencies and proportions for categorical variables. Mean number of OCS dispensings, OCS bursts (defined as a pharmacy claim for an OCS medication with 2 to 28 days’ supply and an average daily dose of ≥20 mg prednisone equivalent), NP-related annual OCS dose, treatment, and exacerbations; sinus surgeries; NP-related HCRU and health care costs were described per patient per year (PPPY) using mean, SD, and median for the pre- and post-mepolizumab periods. Rates before and after mepolizumab initiation were compared by rate ratios, 95% confidence intervals (CIs), and *P* values obtained from generalized estimating equation Poisson regression models with robust standard errors to account for correlation between the pre- and post-mepolizumab periods. Proportions before and after mepolizumab were compared by risk ratios, 95% CIs, and *P* values derived from generalized estimating equation log-binomial regression models.

### Data-sharing statement

Data are owned by Komodo Research database and were accessed by GSK to address the prespecified research questions only. The data are available via a Komodo data license.

## Results

### Patient population

Baseline demographics and characteristics were similar between the overall study population (N = 240) and the on-label subgroup (n = 67). In the overall population and the on-label subgroup, the mean (SD) age was 49.1 (12.2) years and 49.8 (11.4) years; 132 (55%) and 32 (48%) patients were female; and 166 (69%) and 44 (66%) patients had a commercial insurance plan, respectively (Table I). The most common comorbidities included allergic rhinitis, mild/moderate asthma, acute upper respiratory infections, and hyperlipidemia (Table I). The most common prescribers of mepolizumab at index were allergists in the overall population (32%) and otolaryngologists in the on-label subgroup (36%; Table I). Previous receipt of biologics (dupilumab or omalizumab)—that is, during the preindex period—was reported for 17% of patients in the overall population (9%, on-label subgroup); patients receiving biologics were considered to have switched to mepolizumab at the index date. Patient attrition is displayed in Fig E1 in the Online Repository available at www.jaci-global.org. Baseline demographics and characteristics of the severe asthma subgroup are presented in Table E1 in the Online Repository. The median number of claims for mepolizumab after its initiation was 9.4 PPPY and 10.5 PPPY for the overall and on-label populations, respectively.

**Table I:** Demographic and clinical characteristics of patients in overall population and on-label subgroup prescribed INCS

Characteristic	Overall population	On-label subgroup
Sample size	240	67
Demographics∗		
Age (years), mean ± SD [median]	49.1 ± 12.2 [50]	49.8 ± 11.4 [51.3]
Female	132 (55.0)	32 (47.8)
Insurance plan type		
Commercial	166 (69.2)	44 (65.7)
Medicaid	54 (22.5)	13 (19.4)
Medicare	15 (6.3)	7 (10.4)
Unknown	5 (2.1)	3 (4.5)
Race/ethnicity		
Non-Hispanic White	87 (36.3)	20 (29.9)
Non-Hispanic Black	20 (8.3)	6 (9.0)
Hispanic	19 (7.9)	8 (11.9)
Non-Hispanic Asian	15 (6.3)	5 (7.5)
Other	15 (6.3)	4 (6.0)
Unknown	84 (35.0)	24 (35.8)
Year of index date†		
2021	64 (26.7)	12 (17.9)
2022	176 (73.3)	55 (82.1)
Prescriber physician specialty at index date∗‡		
Allergist	77 (32.1)	14 (20.9)
Otolaryngologist	67 (27.9)	24 (35.8)
General practitioner	27 (11.3)	11 (16.4)
Pulmonologist	15 (6.3)	3 (4.5)
Other§	44 (18.3)	12 (17.9)
Unknown	10 (4.2)	3 (4.5)
Quan-CCI,¶‖ mean ± SD [median]	1.1 ± 1.0 [1.0]	1.0 ± 1.0 [1.0]
Length of observation period (months),‖ mean ± SD [median]	12.8 ± 4.2 [13.0]	12.6 ± 3.6 [12.8]
Comorbidities		
Allergic rhinitis	180 (75.0)	42 (62.7)
Asthma (nonsevere)	177 (73.8)	46 (68.7)
Acute upper respiratory infections	103 (42.9)	35 (52.2)
Hyperlipidemia	104 (43.3)	25 (37.3)
COPD	29 (12.1)	9 (13.4)
EGPA	7 (2.9)	3 (4.5)
HES	5 (2.1)	3 (4.5)
Biologics receipt∗∗	41 (17.1)	6 (9.0)
Dupilumab	37 (15.4)	6 (9.0)
Omalizumab	6 (2.5)	1 (1.5)

Data are presented as nos. (%) unless otherwise indicated. *COPD,* Chronic obstructive pulmonary disease; *EGPA,* eosinophilic granulomatosis with polyangiitis; *HES,* hypereosinophilic syndrome; *ICS,* intranasal corticosteroids; *Quan-CCI,* Quan-Charlson Comorbidity Index.

∗ Evaluated on index date.

† ^D^efined as first mepolizumab dispensing after July 1, 2021.

‡ Physician specialty identified via mepolizumab claim.

§ ^”^Other” includes hospital physicians, psychiatrists, and trainees.

¶ Assessed over 12-month baseline, excluding index date.

‖ Observation period spanned from index date to end of eligibility/data.

∗∗ Previous biologic receipt considered switched at index.

### NP-related OCS

**Fig 1:**
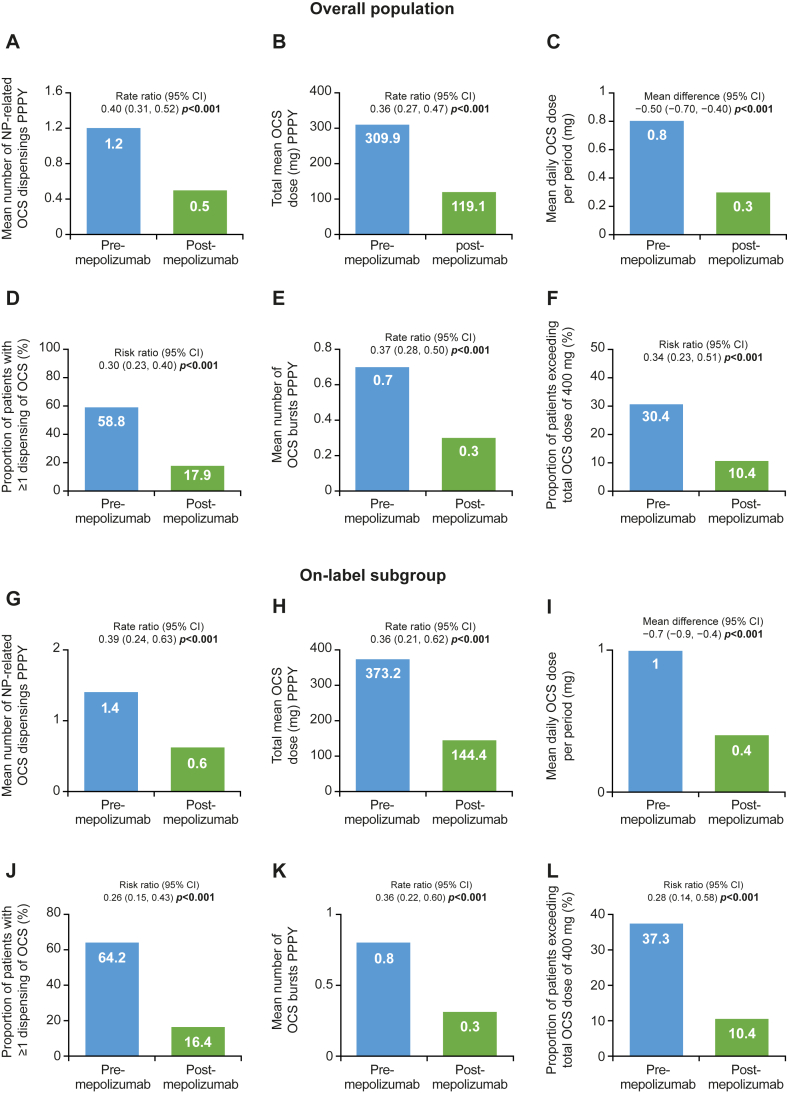
NP-related OCS receipt in overall population **(A-F)** and on-label subgroup prescribed *INCS***(G-L).** OCS burst was calculated as pharmacy claim for OCS medication with 2 to 28 days’ supply and average daily dose of ≥20 mg prednisone (or equivalent). If ≥2 bursts were observed for patient within 14 days, they were considered one burst. *Bold P* values indicate *P* < .05.

NP-related OCS burden was significantly reduced after mepolizumab initiation in the overall population and the on-label subgroup (Fig 1). There was a large improvement among all the NP-related OCS outcomes before versus after mepolizumab, including a reduction in the total mean OCS dose (309.9 mg vs 119.1 mg, respectively; rate ratio [95% CI] 0.36 [0.27, 0.47], *P* < .001, Fig 1, *B*) and the proportion of patients with ≥1 dispensing of OCS (58.8% vs 17.9%, respectively; risk ratio [95% CI] 0.30 [0.23, 0.40], *P* < .001, Fig 1, *D*). Additionally, the mean number of NP-related OCS bursts reduced significantly by >50% after initiation of mepolizumab (0.7 PPPY vs 0.3 PPPY, *P* < .001 respectively; Fig 1, *E*). NP-related OCS burden similarly reduced after mepolizumab initiation for the severe asthma subgroup (see Fig E2 in the Online Repository available at www.jaci-global.org).

### NP-related exacerbations

**Fig 2:**
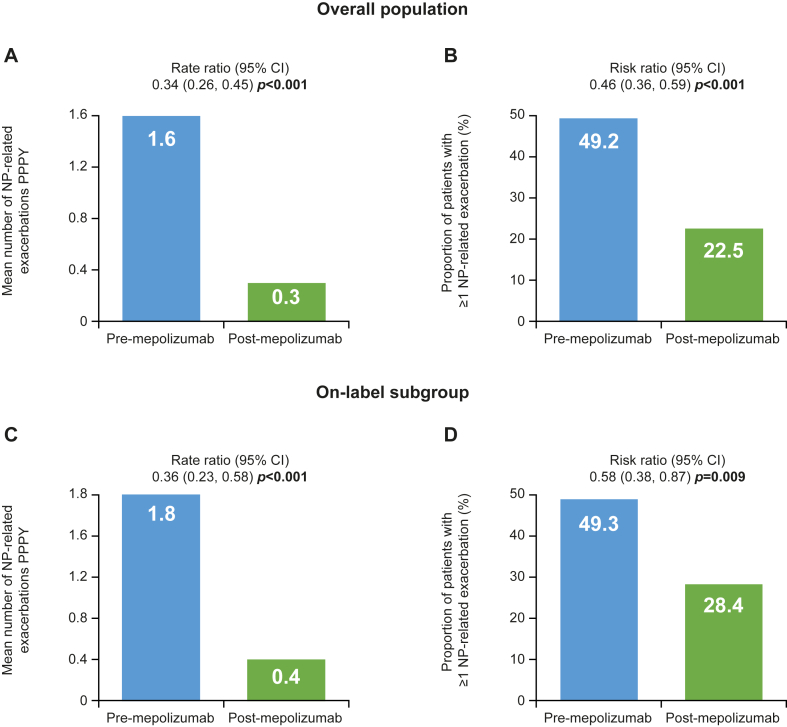
NP-related exacerbations in overall population (**A** and **B**) and on-label subgroup prescribed INCS (**C** and **D**). (*A* and *C*) Mean number of NP-related exacerbations experienced PPPY. (*B* and *D*) Proportion of patients experiencing ≥1 NP-related exacerbations before and after mepolizumab initiation. *Bold P* values indicate *P* < .05.

After initiating mepolizumab, patients experienced a significantly lower mean number of NP-related exacerbations in the overall population, from 1.6 PPPY before to 0.3 PPPY after mepolizumab (rate ratio [95% CI] 0.34 [0.26, 0.45], *P* < .001). There was also a significant reduction in the proportion of patients experiencing ≥1 exacerbation compared with the pre-mepolizumab period (risk ratio [95% CI] 0.46 [0.36, 0.59], *P* < .001) (Fig 2, *A* and *B*). For the on-label subgroup, the mean number of NP-related exacerbations was also significantly reduced after mepolizumab initiation (Fig 2, *C* and *D*). Reductions were also observed in the severe asthma subgroup (see Fig E3 in the Online Repository available at www.jaci-global.org).

### NP-related treatments

The proportion of patients receiving NP-related treatment, including NP-related antibiotics, was significantly reduced after mepolizumab initiation in the overall population (86.7% pre, 75% post; risk ratio [95% CI] 0.87 [0.81, 0.93], *P* < .001). A similar significant reduction of 13% reduction was seen for the on-label subgroup (risk ratio [95% CI] 0.87 [0.79, 0.95], *P* = .003) after mepolizumab initiation. A similar reduction of 9% in the proportion of patients receiving NP-related treatment was observed for the severe asthma subgroup (risk ratio [95% CI] 0.91 [0.85, 0.97], *P* = .008).

### Sinus surgeries

**Fig 3:**
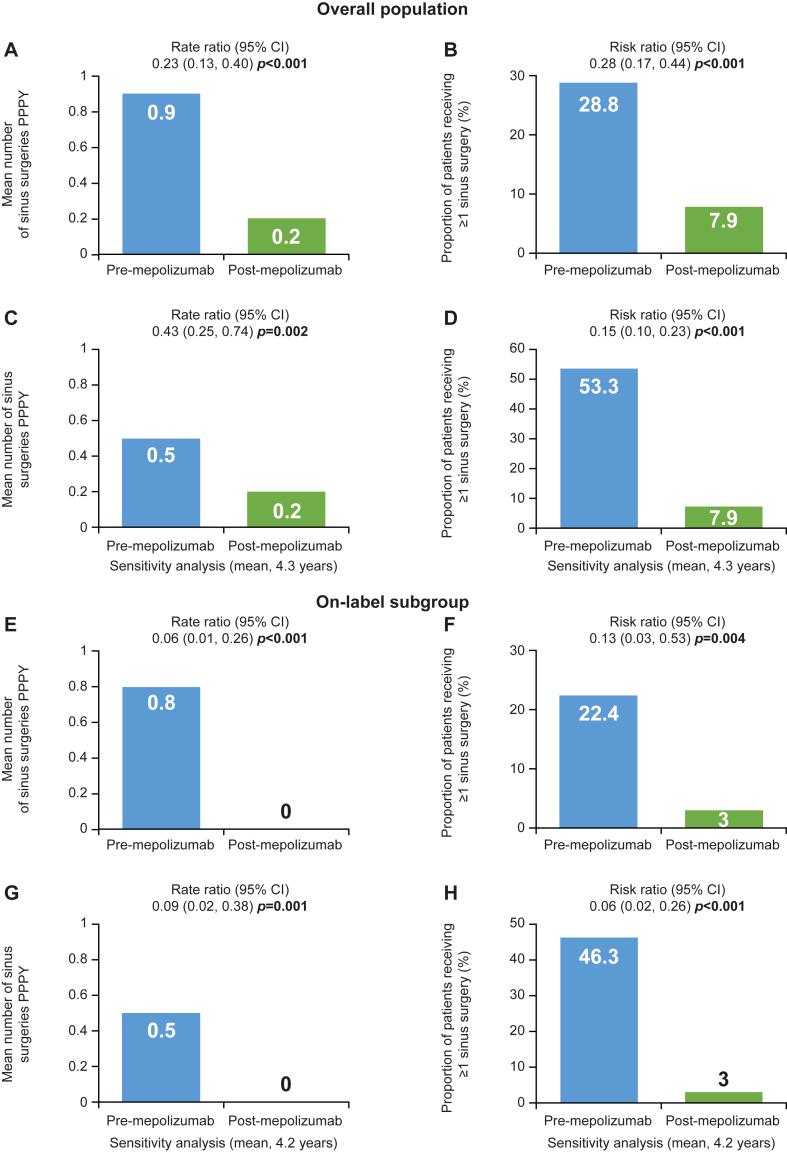
Sinus surgeries in overall population **(A-D)** and on-label subgroup prescribed INCS **(E-H).** (*A* and *B*) Overall population, pre-mepolizumab 1-year lookback. (*C* and *D*) Pre-mepolizumab 4.3-year lookback. (*E* and *F*) On-label subgroup, pre-mepolizumab 1-year lookback. (*G* and *H*) Pre-mepolizumab mean 4.2-year lookback. *Bold P* values indicate *P* < .05.

In the overall population, a significant reduction in the mean number of sinus surgeries was observed after versus before mepolizumab initiation (rate ratio [95% CI] 0.23 [0.13, 0.40], *P* < .001) (Fig 3, *A*). A similar reduction was observed in the sensitivity analysis using all data available before mepolizumab initiation (mean, 4.3 years) (Fig 3, *C*). In the on-label subgroup, significant reductions in the number of sinus surgeries and the proportion of patients receiving ≥1 sinus surgery were also observed (Fig 3, *E-H*). Significant reductions were also seen in the severe asthma subgroup (see Fig E4 in the Online Repository available at www.jaci-global.org).

### NP-related HCRU

**Fig 4:**
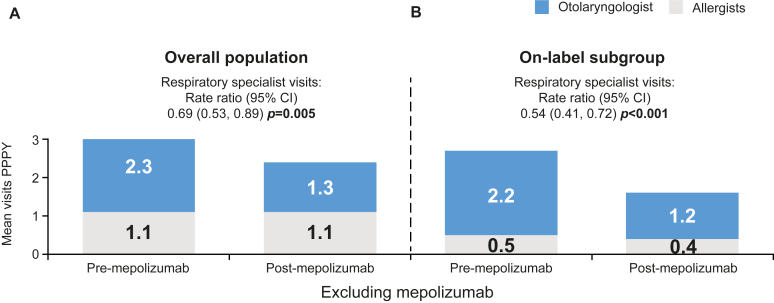
NP-related respiratory specialist visits excluding visits associated with mepolizumab administration before and after mepolizumab initiation for **(A)** overall population and **(B)** on-label subgroup prescribed INCS. Equivalent data, including visits associated with mepolizumab administration, are presented in Table E2. *Bold P* values indicate *P* < .05.

For the overall population and on-label subgroup, a significant reduction of ≥30% in the number of NP-related respiratory specialists visits (allergist/immunologist and otolaryngologist visits) was observed, excluding visits associated with the administration of mepolizumab. In the overall population, significant reductions in the number of NP-related otolaryngologist visits PPPY were observed after mepolizumab initiation (Fig 4, *A*) (rate ratio [95% CI] 0.52 [0.43, 0.63], *P* < .001), excluding visits associated with mepolizumab administration. For the on-label subgroup, significant reductions were observed for respiratory specialist visits and otolaryngologist visits (*P* < .001), excluding visits associated with mepolizumab administration (Fig 4, *B*). NP-related HCRU for the severe asthma subgroup is presented in Fig E5 in the Online Repository available at www.jaci-global.org. Additionally, NP-related HCRU including visits associated with mepolizumab administration are presented in Table E2, also in the Online Repository.

### NP-related health care costs

**Fig 5:**
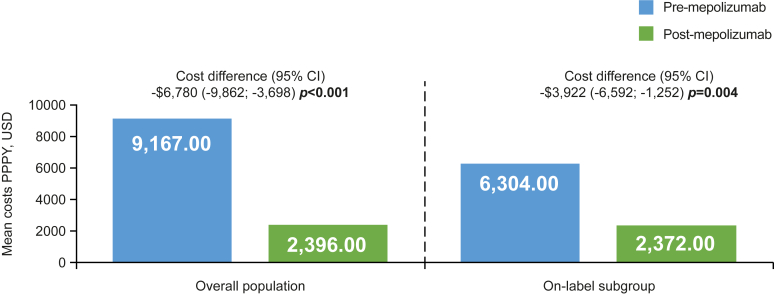
NP-related total medical and pharmacy costs, including cost associated with mepolizumab administration before and after therapy initiation. NP-related health care costs were defined as any medical visits with primary or secondary diagnosis of NP or NP surgery; NP-related pharmacy costs (USD) were defined as any claim associated with NP-related treatment. Equivalent data excluding costs associated with mepolizumab administration are presented in Fig E7. *Bold P* values indicate *P* < .05. *USD,* US dollar.

**Fig 6:**
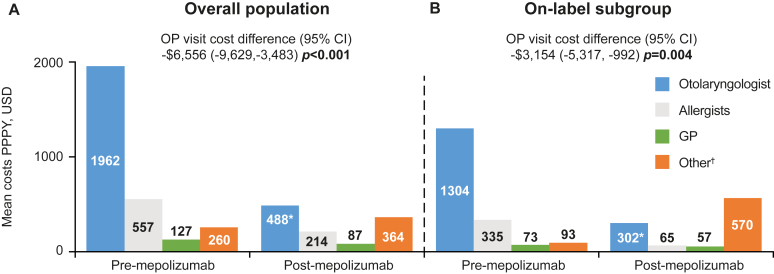
NP-related OP costs before and after mepolizumab initiation including costs associated with mepolizumab administration for **(A)** overall population and **(B)** on-label subgroup prescribed INCS. ∗*P* < .05. †Other OP visits included OP specialties other than allergist, otolaryngologist, and GP. Equivalent data excluding costs associated with mepolizumab administration are presented in Fig E8. *Bold P* values indicate *P* < .05. *GP,* General practitioner; *OP,* outpatient; *USD,* US dollar.

Numerically lower total NP-related medical and pharmacy costs (including costs associated with mepolizumab) were observed for patients after mepolizumab initiation in the overall population; this was also seen in the on-label subgroup (Fig 5). Similarly, numerical reductions were also observed in total NP-related medical and pharmacy costs for patients in the severe asthma subgroups (see Fig E6 in the Online Repository available at www.jaci-global.org). When excluding costs associated with mepolizumab, significant reduction in costs was observed for the overall population and on-label subgroup (see Fig E7 in the Online Repository), In the overall population, NP-related outpatient visit costs were numerically lower after versus before mepolizumab initiation, which includes costs associated with, otolaryngologist, allergists/immunologist, general practitioner, and other specialists’ visit costs while including the cost of mepolizumab administration in these settings (Fig 6). Furthermore, mepolizumab initiation was associated with significant reductions in mean costs for otolaryngologist visits for the overall population (Fig 6). Additionally, a significant reduction in costs associated with sinus surgery was observed between the pre- and post-mepolizumab period in the overall population (cost difference [95% CI] −$4,797 [−$7,698, −$1,897]; *P* = .001). Significant reductions were seen for otolaryngologist visits for the on-label subgroup, including costs associated with mepolizumab administration (cost difference [95% CI] −$855 [−1,519, −252]; *P* = .006) (Fig 6). In addition, there were reductions in outpatient costs for the severe asthma subgroup. NP-related costs, excluding costs associated with mepolizumab administration, are presented in Figs E7, E8, and E9 in the Online Repository available at www.jaci-global.org.

### Composite end points

**Fig 7:**
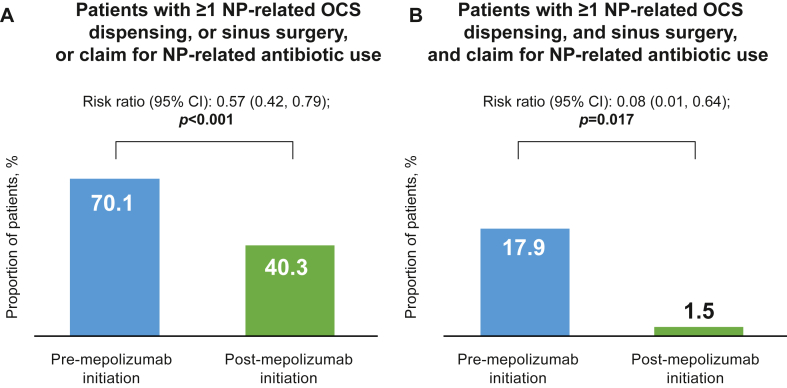
Proportion of patients meeting composite end points. **(A)** ≥1 NP-related OCS dispensing or sinus surgery or NP-related antibiotic claim. **(B)** ≥1 NP-related OCS dispensing and sinus surgery and NP-related antibiotic claim before and after mepolizumab initiation (on-label subgroup prescribed INCS). *Bold P* values indicate *P* < .05. *OP,* Outpatient.

In the on-label subgroup, there was a significant reduction of in the proportion of patients meeting both composite end points of ≥1 NP-related OCS dispensing or sinus surgery or claim for NP-related antibiotic receipt (−43%, *P* < .001) and ≥1 NP-related OCS dispensing, and sinus surgery and claim for NP-related antibiotic receipt (−92%, *P* = .017) after versus before mepolizumab initiation (17.9%) (Fig 7).

## Discussion

In this retrospective study assessing the impact of mepolizumab treatment for patients with CRSwNP, mepolizumab significantly improved all clinical outcomes, which included reductions in NP-related OCS and treatment burden, as well as number of NP-related exacerbations, sinus surgeries, and respiratory specialist visits, notably otolaryngologist visits. Similar results were observed across the overall, on-label, and severe asthma subgroups, including significant reductions in clinical outcomes and trends toward reductions in HCRU and health care costs.

These results are comparable with those observed in the phase 3 SYNAPSE trial, which demonstrated the efficacy and safety of mepolizumab for patients with severe CRSwNP, including those with comorbid asthma.^3^ NP-related OCS receipt reduced after mepolizumab initiation in both studies; in SYNAPSE, 25% of patients receiving mepolizumab required ≥1 course of SCS for CRSwNP compared with 37% of patients receiving placebo (*P* = .02), while in the current study the proportion of patients receiving ≥1 OCS dispensing fell from 58.8% before mepolizumab treatment to 17.9% after mepolizumab treatment (*P* < .001). Similar consistent results were observed in sinus surgeries. In SYNAPSE, only 9% of patients receiving mepolizumab underwent nasal surgery compared with 23% of patients receiving placebo (*P* = .003); a significant reduction in sinus surgery after mepolizumab was also observed in this study (28.8% pre vs 7.9% post, *P* < .001). These improvements are reflected in the subsequent lowering of HCRU reported here, further substantiating the benefits of mepolizumab for patients with CRSwNP. The current findings should be considered robust because outcomes were evaluated among an overall population, an on-label subgroup, and in patients with comorbid severe asthma, all broadly supporting the initiation of mepolizumab in this patient population, as substantiated by the findings of SYNAPSE.^3^

This study was to our knowledge the first retrospective claims-based database study focused specifically on CRSwNP-related outcomes, and also excluded patients without comorbid severe asthma. This was implemented to help ensure the population under investigation was being treated for CRSwNP rather than for severe asthma (or severe asthma accompanied by CRSwNP), with the aim of providing insight on the efficacy of mepolizumab at improving NP-related outcomes for patients with comorbid mild asthma in clinical practice. These positive results suggest that the treatment benefits of mepolizumab are CRSwNP-specific and not secondary benefits only, from improved asthma control. As previously noted, similar results were reported for the overall population and on-label subgroup (which included patients being prescribed INCS), which is encouraging regarding the potential benefit associated with mepolizumab in practice.

The inclusion of patients with multiple comorbidities, such as comorbid asthma and allergic rhinitis, reflects an in-practice clinical population and enhances the generalizability of the study results. This broad eligibility contrasts with phase 3 efficacy trials with strict enrollment criteria. Another strength of this study included the pre- and post-mepolizumab study design, resulting in each patient serving as his or her own control, thus differing from studies versus placebo, where patient variances between demographics and characteristics are encountered between treatment groups (although these are mitigated to an extent by randomization), which may introduce residual confounding. While placebo-controlled studies are still the gold standard, retrospective evidence—reflective of real-world practice—offers additional practice-based insights into the effectiveness of a drug beyond the confines of a clinical trial setting.

The following study limitations should be considered. There was no washout period before mepolizumab administration, so it is not possible to determine that the effects seen during the observation period are attributable to mepolizumab alone or to the other nonexclusionary biologics also indicated for CRSwNP (ie, dupilumab and omalizumab) administered during the pre-mepolizumab period. It should also be noted that a washout period is not typically undertaken except when considered necessary to achieve prior authorization/insurance approval and delivery/administration for the next choice of biologic therapy. The decision to include patients treated with dupilumab or omalizumab at baseline was to reflect treatment patterns seen in practice for patients with CRSwNP. Furthermore, because reslizumab, benralizumab, and tezepelumab are biologics approved for severe asthma and not CRSwNP, the decision to exclude patients receiving these drugs supported the selection of the specified patient population. Ultimately, the proportion of patients who received dupilumab or omalizumab in the pre-mepolizumab period was low (15.4% and 2.5%, respectively). Additionally, the data presented are before and after mepolizumab administration and cannot capture whether patients took their medication as prescribed or whether they had used any add-on over-the-counter medication. Although in clinical practice treatment adherence is largely assumed with 6 to 7 mepolizumab administrations per year, it should be noted that adherence may have not been optimal for all patients, but this reflects real-world practice. As with all database studies, the data collected may have been vulnerable to coding inaccuracies, which may have led to misclassification bias. Last, health care costs reported here both exclude and include costs associated with the administration of mepolizumab. While trends toward reductions in health care costs were seen, including mepolizumab administration, it is not clear whether these effects would be demonstrated for longer time horizons, which would accrue additional costs of monthly mepolizumab administration.

To our knowledge, ours is the first retrospective study to assess mepolizumab effectiveness in patients with CRSwNP without comorbid severe asthma on CRSwNP-specific outcomes. The study design utilized comparison to the pre-mepolizumab period, which mirrors visit-to-visit assessments as done by respiratory specialists in practice settings when treating patients with CRSwNP. Significant reductions were observed across all outcomes between the pre and post-mepolizumab periods, particularly highlighting effectiveness for outcomes that resonate with practitioners and patients alike, such as steroid reduction, mitigation of NP exacerbations, and surgical benefit. In summary, mepolizumab was found to improve treatment resource utilization burden for patients with CRSwNP, exemplified in clinical practice.

## Disclosure statement

Funded by GSK (ID 218957). The sponsor was involved in study design and implementation, as well as data collection, analysis, interpretation, and writing and reviewing the study report. The sponsor did not place any restrictions on the authors’ access to data or statements made in the report. All authors had full access to study data and had the final responsibility for the decision to submit for publication.

Data-sharing statement: Data are owned by Komodo Research database and were accessed by GSK to address the prespecified research questions only. The data are available via Komodo data license.

Disclosure of potential conflict of interest: J. C. Cardet reports receiving honoraria from Aiolosbio, Amgen, Apogee, 10.13039/100004325AstraZeneca, Chiesi, GSK, Genentech, and Sanofi for work on advisory boards and steering committees, and for educational lectures; and funding from the National Heart, Lung and Blood Institute (R21HL172124), the 10.13039/100002590American Lung Association/10.13039/100002567American Academy of Allergy, Asthma & Immunology Allergic Respiratory Diseases Award (AI-835475), a GSK investigator-initiated study grant, and the 10.13039/100001009Bristol Myers Squibb Foundation Winn Award. J. Silver was formerly employed by GSK and holds financial equities in GSK and is now employed by Amgen and holds financial equities in Amgen. F. Laliberté, C. Gao, R. Ramasubramanian, A. Hilts, and K. Zhang are employees of Analysis Group, a consulting company that received payment from GSK to conduct this study but did not receive payment for the report’s development. J. Hwee, W. Ahmed, and A. G. Edgecomb are employed by GSK and hold financial equities in GSK. M. Maldonado-Puebla declares no relevant conflicts of interest.
