# Supplementary material for: Mepolizumab for the management of chronic rhinosinusitis with nasal polyps across the United States: A retrospective study
Source: J Allergy Clin Immunol Glob. 2025 Jul 31;4(4):100549. doi: 10.1016/j.jacig.2025.100549 (PMC12444170; doi:10.1016/j.jacig.2025.100549)
Supplement: Supplementary Data [file mmc1.docx]

## **Supplementary tables/figures**

### Table E1. Demographics and clinical characteristics of patients with comorbid severe asthma

| **Characteristics** | **Severe asthma subgroup** |
| --- | --- |
|  | **N=125** |
| **Demographics^1^** |  |
| Age, years, mean ± SD [median] | 50.7 ± 12.4 [53.0] |
| Female, n (%) | 84 (67.2) |
| Insurance plan type, n (%) | - |
| Commercial | 74 (59.2) |
| Medicaid | 37 (29.6) |
| Medicare | 8 (6.4) |
| Unknown | 6 (4.8) |
| Race/ethnicity, n (%) | - |
| Non-Hispanic White | 41 (32.8) |
| Non-Hispanic Black | 19 (15.2) |
| Hispanic | 12 (9.6) |
| Non-Hispanic Asian | 7 (5.6) |
| Other | 9 (7.2) |
| Unknown | 37 (29.6) |
| **Year of index date,^2^ n (%)** | **-** |
| 2021 | 38 (30.4) |
| 2022 | 87 (69.6) |
| 2023 | NA |
| **Prescriber physician specialty at the index date,^1,3^n (%)** | - |
| Allergist | 51 (40.8) |
| Otolaryngologist | 11 (8.8) |
| General practitioner | 11 (8.8) |
| Pulmonologist | 19 (15.2) |
| Other^4^ | 24 (19.2) |
| Unknown | 9 (7.2) |
| **Quan-CCI,^5,6,^ mean ± SD [median]** | 1.5 ± 1.1 [1.0] |
| **Length of observation period (months),^6^ mean ± SD [median]** | 12.2 ± 4.6 [11.0] |
| **Comorbidities, n (%)** | - |
| Allergic rhinitis | 102 (81.6) |
| Asthma | 124 (99.2) |
| Acute upper respiratory infections | 61 (48.8) |
| Hyperlipidemia | 44 (35.2) |
| COPD | 32 (25.6) |
| EGPA | 6 (4.8) |
| HES | 6 (4.8) |
| **Biologics use^7^, n (%)** | 22 (17.6) |
| Dupilumab | 16 (12.8) |
| Omalizumab | 6 (4.8) |

^1^Evaluated on index date; ^2^defined as the first mepolizumab dispensing after July 1, 2021; ^3^Physician specialty identified via mepolizumab claim; ^4^“Other” includes hospital physicians, psychiatrists, and trainees; ^5^Assessed over a 12-month baseline, excluding index date; ^6^Observation period spanned from index date to end of eligibility/data; ^7^Previous biologic users considered switched at index.

COPD, chronic obstructive pulmonary disease; EGPA, eosinophilic granulomatosis with polyangiitis; HES, hypereosinophilic syndrome; INCS, intranasal corticosteroids; Quan-CCI, Quan-Charlson Comorbidity Index; SD, standard deviation.

### Table E2. NP-related HCRU and costs including visits and costs associated with mepolizumab administration, pre- and post-mepolizumab initiation

|  | **Subgroup** | | |
| --- | --- | --- | --- |
| **HCRU** | **Overall population** | **On-label subgroup** | **Severe asthma subgroup** |
|  | **N=240** | **N=67** | **N=125** |
| Respiratory specialist visits, mean PPPY |  |  |  |
| Pre-mepolizumab | 3.4 | 2.7 | 3.1 |
| Post-mepolizumab | 3.4 | 2.3 | 2.9 |
| Rate ratio (95% CI) | 0.99 (0.79, 1.23) | 0.84 (0.58, 1.21) | 0.89 (0.72, 1.08) |
| P-value | 0.899 | 0.347 | 0.240 |
| Allergist visits, mean PPPY |  |  |  |
| Pre-mepolizumab | 1.1 | 0.5 | 1.2 |
| Post-mepolizumab | 1.8 | 0.9 | 1.6 |
| Rate ratio (95% CI) | 1.74 (1.17, 2.59) | 1.92 (0.95, 3.87) | 1.24 (0.92, 1.68) |
| P-value | **0.006** | 0.068 | 0.160 |
| Otolaryngologist visits, mean PPPY |  |  |  |
| Pre-mepolizumab | 2.3 | 2.2 | 1.9 |
| Post-mepolizumab | 1.5 | 1.4 | 1.3 |
| Rate ratio (95% CI) | 0.63 (0.52, 0.76) | 0.59 (0.43, 0.81) | 0.66 (0.51, 0.86) |
| P-value | **<0.001** | **0.001** | **0.002** |
| **#** |  | | |
|  |  |  |  |
|  |  |  |  |
|  |  |  |  |
|  |  |  |  |
|  |  |  |  |
|  |  |  |  |
|  |  |  |  |
|  |  |  |  |
|  |  |  |  |
|  |  |  |  |

*CI, confidence interval; HCRU, healthcare resource utilization; NP, nasal polyps; PPPY, per patient per year; SD, standard deviation; USD, United States dollar*

### Figure E1. Patient attrition


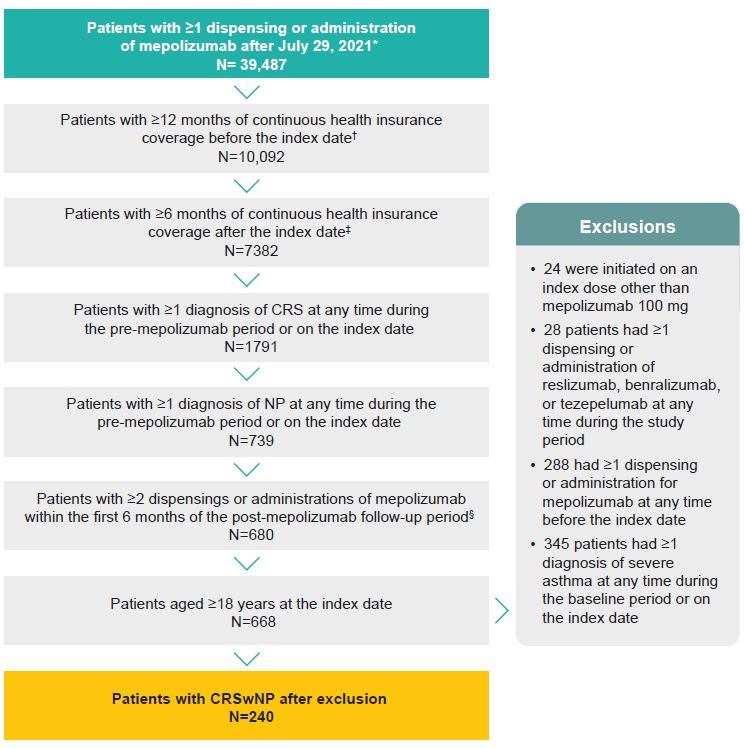


### Figure E2. NP-related OCS use in patients with comorbid severe asthma


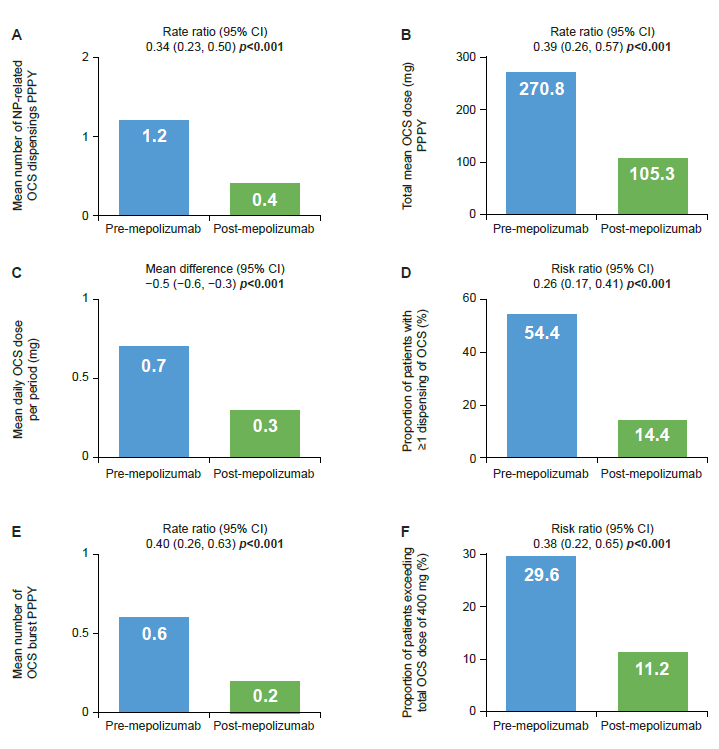


*An OCS burst was calculated as a pharmacy claim for an OCS medication with 2–28 days supply and an average daily dose of ≥20 mg prednisone (or equivalent). If ≥2 bursts were observed for a patient within 14 days of each other, they were considered one burst.* p-values are in bold text where p<0.05.

*CI, confidence interval;NP, nasal polyps;OCS, oral corticosteroids;PPPY, per patient per year.*

###
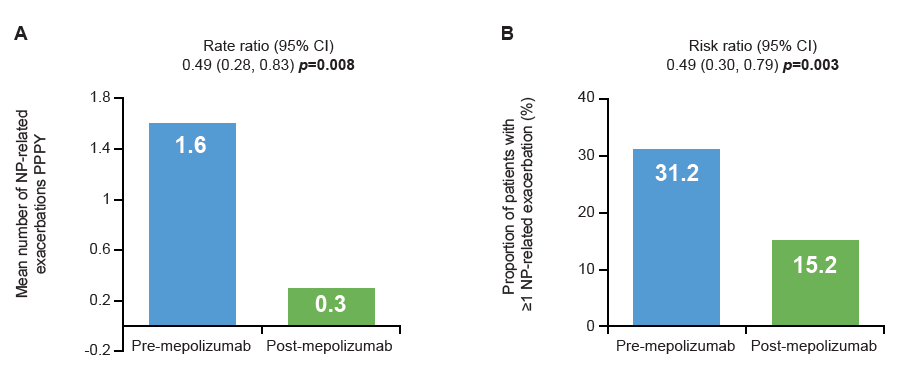
Figure E3. NP-related exacerbations in patients with comorbid severe asthma

(A) Mean number of NP-related exacerbations experienced PPPY (B) Proportion of patients experiencing ≥1 NP-related exacerbations pre- and post-mepolizumab initiation.

p-values are in bold text where p<0.05.

*CI, confidence interval; PPPY, per patient per year.*

###
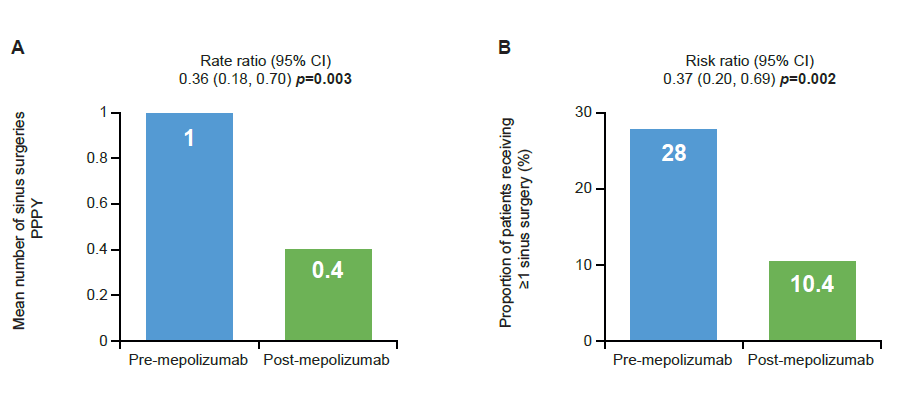
Figure E4. Sinus surgeries in patients with comorbid severe asthma

Pre-mepolizumab, 1-year lookback: (A) Mean number of sinus surgeries PPPY, (B) Proportion of patients receiving ≥1 sinus surgery.

p-values are in bold text where p<0.05.

*CI, confidence interval; PPPY, per patient per year.*

###
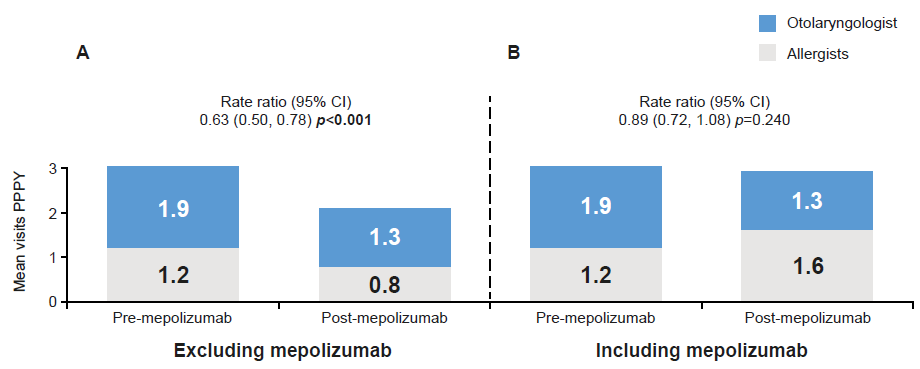
Figure E5. NP-related respiratory specialist visits (A) Excluding visits associated with mepolizumab administration pre- and post-mepolizumab initiation and (B) Including visits associated with mepolizumab administration pre- and post-mepolizumab initiation for patients with comorbid severe asthma

p-values are in bold text where p<0.05.

*CI, confidence interval; PPPY, per patient per year.*

### Figure E6. NP-related total medical and pharmacy costs including costs associated with mepolizumab administration pre- and post-mepolizumab initiation in patients with comorbid severe asthma


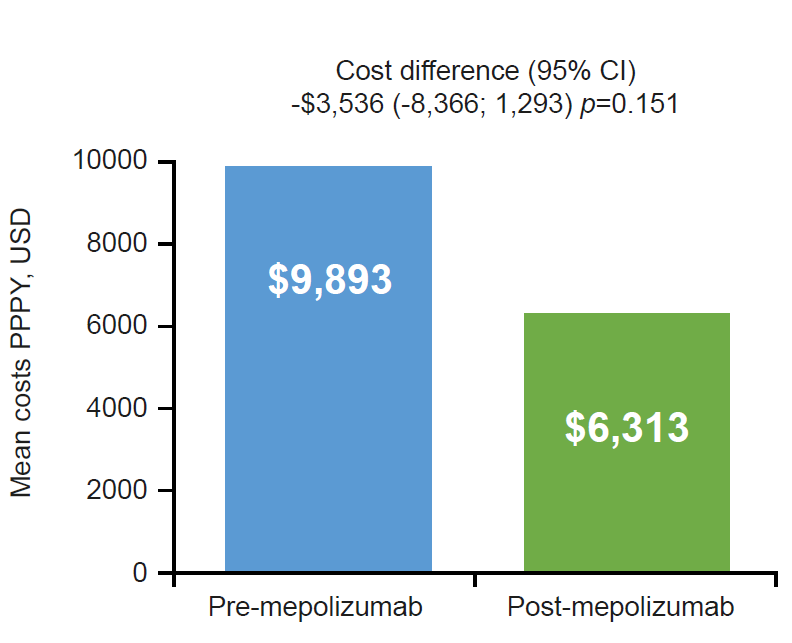


Healthcare costs were defined as any medical visits with a primary or secondary diagnosis of NP or an NP surgery; NP-related pharmacy costs were defined as any claim associated with an NP-related treatment – reported in US dollars.

*CI, confidence interval; PPPY, per patient per year; USD, United States dollar.*

### Figure E7. NP-related total medical and pharmacy costs, excluding cost associated with mepolizumab administration, pre- and post-mepolizumab initiation
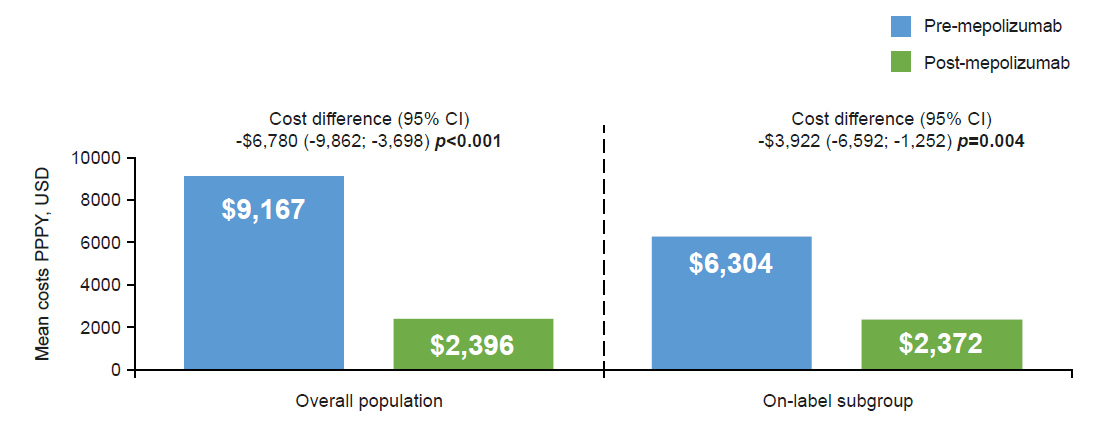


NP-related healthcare costs were defined as any medical visits with a primary or secondary diagnosis of NP or an NP surgery; NP-related pharmacy costs (US dollars) were defined as any claim associated with an NP-related treatment. p-values are in bold text where p<0.05.

*CI, confidence interval; PPPY, per patient per year; USD, United States dollar.*

### Figure E8. NP-related OP costs pre- and post-mepolizumab initiation excluding costs associated with mepolizumab administration for the (A) Overall population and (B) On-label subgroup prescribed INCS

*
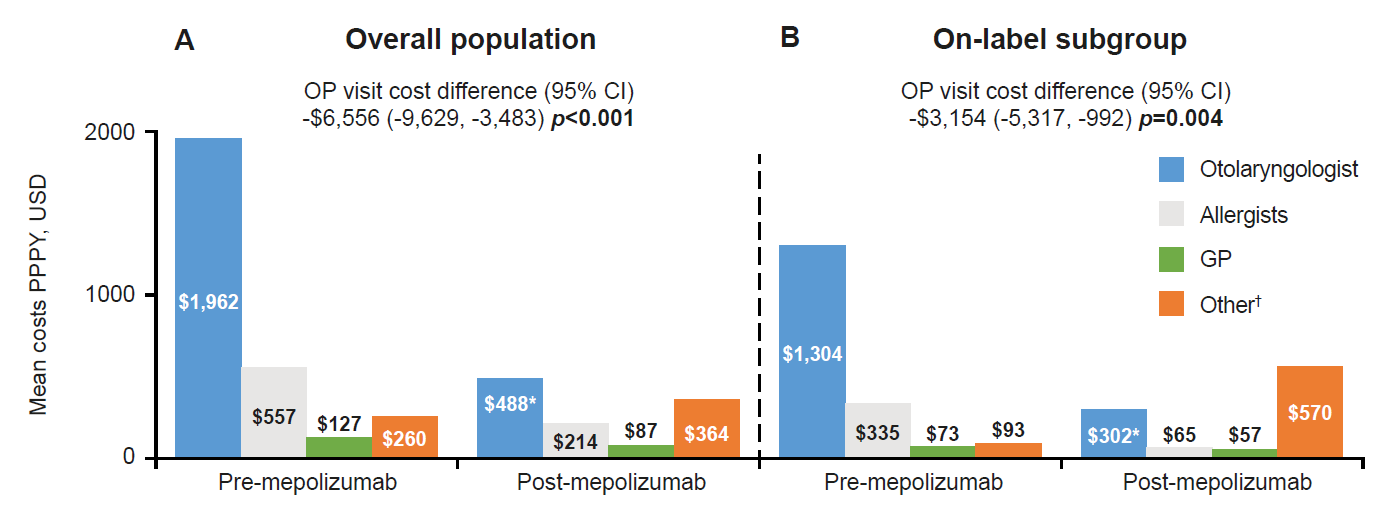
*

*p<0.001 ^†^other OP visits included OP specialties other than allergist, otolaryngologist, and GP. p-values are in bold text where p<0.05.

*CI, confidence interval; GP, general practitioner;* *INCS, intranasal corticosteroids; OP, outpatient; PPPY, per patient per year; USD, United States dollar.*

###
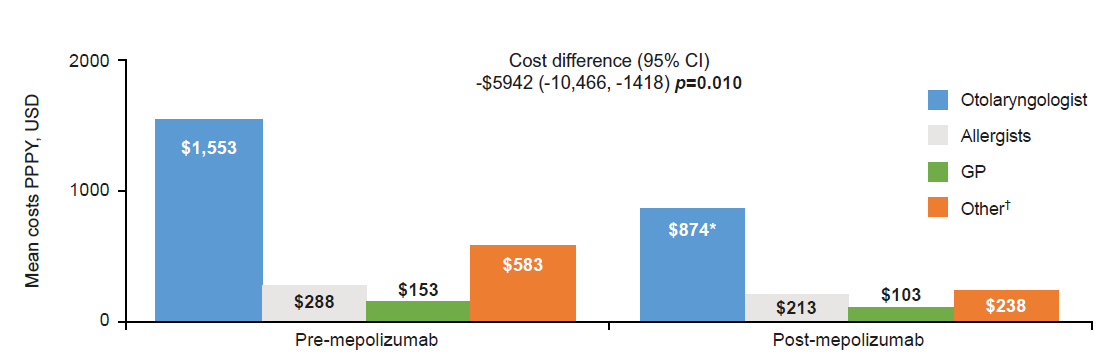
Figure E9. NP-related OP costs pre- and post-mepolizumab initiation excluding costs associated with mepolizumab administration for patients with comorbid severe asthma

*p<0.1; ^†^other OP visits included OP specialties other than allergist, otolaryngologist, and GP.

*CI, confidence interval; GP, general practitioner*; *OP, outpatient; PPPY, per patient per year; USD, United States dollar.*
